# GCN250, new global gridded curve numbers for hydrologic modeling and design

**DOI:** 10.1038/s41597-019-0155-x

**Published:** 2019-08-12

**Authors:** Hadi H. Jaafar, Farah A. Ahmad, Naji El Beyrouthy

**Affiliations:** American University of Beirut, Beirut, Lebanon

**Keywords:** Climate and Earth system modelling, Hydrology, Natural hazards, Ecological modelling

## Abstract

The USDA curve-number (CN) method is fundamental for rainfall-runoff modeling. A global CN database is not currently available for geospatial hydrologic analysis at a resolution higher than 0.1°. We developed a globally consistent, gridded dataset defining CNs at the 250 m spatial resolution from new global land cover (300 m) and soils data (250 m). The resulting data product – GCN250 – represents runoff for a combination of the European space agency global land cover dataset for 2015 (ESA CCI-LC) resampled to 250 m and geo-registered with the hydrologic soil group global data product (HYSOGs250m) released in 2018. Our analysis indicated that medium to high runoff potential currently dominates the globe, with curve numbers ranging between 75 and 85. Global curve numbers were 62, 78, and 90 for dry, average, and wet antecedent runoff conditions, respectively. Australia has the highest runoff potential, while Europe has the lowest. Runoff ratios compare well with GLDAS. The potential application of this data includes hydrologic design, land management applications, flood risk assessment, and groundwater recharge modeling.

## Abstract (summary)

**Table:** 

Design Type(s)	modeling and simulation objective • data integration objective
Measurement Type(s)	hydrological process
Technology Type(s)	computational modeling technique
Factor Type(s)	geographic location • hydrological process • soil • land use
Sample Characteristic(s)	Earth (Planet) • soil • land • ecosystem • hydrological process

Machine-accessible metadata file describing the reported data (ISA-Tab format)

## Background & Summary

Land cover and soils play a fundamental role in the hydrologic cycle by controlling infiltration and affecting surface and groundwater flows. The Natural Resources Conservation Service (NRCS) of the United States Department of Agriculture (USDA) developed a simple, stable, and predictable method for calculating runoff from rainfall events^1^. Recently and with the increasing availability of routine land cover products, there have been few attempts to develop regional and global curve number datasets. Hong and Adler^2^ generated a global CN dataset at the 0.1° resolution based on (1) the global land cover data from the Moderate Resolution Imaging Spectroradiometer (MODIS) at 1-km resolution produced in 2002^3^ and (2) the Digital Soil Map of the World (DSMW) published in 2003 (100-km resolution) by the Food and Agriculture Organization (FAO) of the United Nations. Hong and Adler^2^ derived the CN values for each hydrological soil group by mapping MODIS land cover classification into the National Engineering Handbook (NEH)^4^ descriptors for land cover under fair hydrological conditions. However, their global CN dataset was not made public. Zeng, *et al*.^5^ used the MODIS 500 m Land Cover product^6^ of 2013 with the Harmonized World Soil Database (HWSD) v.1.2 and the Digital Soil map of the World (DSMW) v3.6 as amended by FAO in 2007 to generate a global CN map at a “fine” resolution, believed to be 500 m (by downscaling the 30 arc-second HWSD data). The Zeng, *et al*.^5^ global CN dataset was also not publicly available. In the two works (Hong and Adler^2^ as well as Zeng, *et al*.^5^), the CN datasets were produced by converting the soil classification in the FAO database to hydrologic soils group (HSG) using the provided soil properties based on the USDA soil texture classification scheme^7^. Ross, *et al*.^8^ generated the first publicly available gridded dataset of HSG at the 250 m resolution (HYSOGs250m) from soil texture, depth to bedrock, and groundwater, also following USDA specifications^7^. The generation of HYSOGs250m data triggered our attempt to create a synergistic curve number product exploiting the most recent land cover (LC) data (2015) at a similar resolution (300 m). The newly released global LC maps for 2015 were developed by the European Space Agency (ESA) Climate Change Initiative Land Cover Project (CCI-LC)^9^. This project produces global annual LC maps starting from the 1990s through 2015 (and beyond) based on several satellite sensors: Advanced Very High Resolution Radiometer (AVHRR), Satellite Pour l’Observation de la Terre Vegetation (SPOT-VGT), Medium Resolution Imaging Spectrometer (MERIS), and Project for On-Board Autonomy – Vegetation (PROBA-V). The annual 2015 ESA CCI-LC map was developed from a baseline LC map by utilizing the entire MERIS archive (2003–2012) and PROBA-V data for 2013–2015. We generated the first global gridded CN dataset (GCN250) from the ESA CCI- LC maps (2015) and the HYSOGs250m soils data based on the USDA curve number tables^4^ and plant functional types^10^. The GCN250 datasets represent the global curve numbers at approximately 250 m spatial resolution under dry, average, and wet antecedent runoff conditions (ARC). The soil was assumed undrained soil, and hence the CN of dual HSG were treated the same as the HSG class D.

We believe that this new CN dataset will be of value and interest to the scientific community because it can be directly used to assess time series changes in runoff at global, regional, and watershed scale, given the availability of a consistent ESA land cover product from 1992–2015^11^. The GCN250 dataset is valuable for hydrological analysis and design, flood risk assessment, and mapping, watershed water management, and other related applications. Rainfall-runoff modeling is a potential application given the available techniques in downscaling gridded precipitation data.

## Methods

**Fig. 1:**
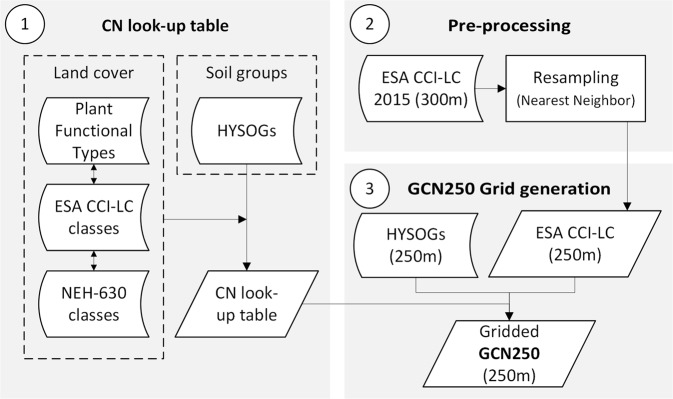
Schematic overview of the generation of global curve number (GCN250) product. Three main steps are illustrated: (1) CN look-up table is created based on mapping ESA CCI-LC land cover classes into National Engineering Handbook – Part 630 Hydrology land cover classes (NEH-630); (2) ESA CCI-LC 2015 300 m raster is resampled to spatial resolution ~250 m (ESA-CCI LC 250 m); (3) CN values (GCN250) are mapped at ~250 m spatial resolution based on the hydrologic soil groups dataset (HYSOGs250m), CN look-up table (step 1), and the resampled ESA CCI-LC 250m raster (step 2).

We used three main inputs to generate the GCN250 datasets (Fig. 1): a land use/land cover map, a hydrologic soil group map, and three CN look-up tables. For the land cover product, we used the most recent ESA-CCI LC data of 2015 (ESA European Space Agency^9^). The hydrologic soil groups were acquired from Ross, *et al*.^8^. The CN look-up table was created based on the USDA Soil Conservation Service (SCS) Runoff Curve Number (CN) method^7^. GCN250 was created within the R open source environment^12^ using the Raster library functions^13^.

### Land cover mapping

CN values were determined by mapping ESA land cover classes (2015) into NEH-630 classes. The ESA land cover classes are classified into various plant functional types (PFT)^10^. The detailed description for each land cover class is provided by ESA European Space Agency^9^ and Poulter, *et al*.^10^. We mapped the PFT into Land cover classes according to NEH-630 classification (Table 1). Because not all plant functions types exist in the NEH-630 classification, we made certain assumptions to make sure that all PFTs of the ESA land cover classes have a corresponding NEH-630 land cover class. Trees PFT were mapped as woods classes in NEH-630, while shrubs in PFT were mapped as desert shrubs and brushes classes in NEH-630. The hydrologic conditions of the wood and shrubs/brushes classes were used to distinguish between the PFT types of trees and shrubs. For example, broadleaf evergreen trees have a lower CN than broadleaf deciduous, and therefore we mapped them as woods with under good hydrologic conditions. Similarly, deciduous needle leaf trees have a higher runoff potential than any other tree type. Therefore, we mapped them as woods under a poor hydrologic condition. We mapped natural grass in the PFT class as the NEH-630 grasslands class under good hydrologic conditions. For managed grasslands (which are rain-fed croplands in ESA classes), we calculated CN values from the average CN of all cropland types in NEH-630 (row crops, small grains, and close-seeded or broadcast legumes or rotation meadow) under good hydrologic conditions. Bare soil CN values were matched with the fallow bare soil.

**Table 1:** Curve number (CN) values derived for hydrological soil groups (A, B, C, and D) for plant functional types (PFT) and their matches in National Engineering Handbook – Part 630 Hydrology land cover classes (NEH-630).

PFT	NEH-630 classes (hydrologic condition)	CN for hydrological soil group			
		A	B	C	D
Tree Broadleaf Evergreen	Woods (good)	30	55	70	77
Tree Broadleaf Deciduous	Woods (average CN of fair and good condition)	33	58	72	78
Tree Needleleaf Evergreen	Woods (average CN of fair and good condition)	33	58	72	78
Tree Needleleaf Deciduous	Woods (poor)	45	66	77	83
Shrub Broadleaf Evergreen	Average CN of Desert shrub (good), and Brush (good)	40	58	72	79
Shrub Broadleaf Deciduous	Average CN of Desert shrub (good), and Brush (fair)	42	62	75	81
Shrub Needleleaf Evergreen	Average CN of Desert shrub (good), and Brush (fair)	42	62	75	81
Shrub Needleleaf Deciduous	Does not exist in ESA LC types	—	—	—	—
Natural grass	Grassland (good)	39	61	74	80
Managed grass (rainfed)	Average CN of good hydrologic conditions for all cropland types	57	68	75	78
Bare soil	Fallow (bare soil)	77	86	91	94
Water (for wetlands only)	—	100	100	100	100

### Composite curve number

Curve numbers used in generating GCN250 are presented in Online-only Table 1 along with the PFT decomposition of each ESA LC classes. CN values for the ESA CCI-LC classes that have multiple PFTs were calculated using a weighted average:

$$C{N}_{k}=\mathop{\sum }\limits_{i=0}^{n}C{N}_{i}\times F{C}_{i}$$

where CN_k_ is the curve number of the class for the hydrological soil group k, n is the total number of PFT types (i) present in the ESA-CCI LC class, CN_i_ is the curve number for the individual PFT class - hydrological soil group k combination and FC_i_ is the percentage of the PFT type i of the total LC class n based on ESA-CCI LC description detailed in the ESA LC product user guide^9^.

Some exceptions are listed hereafter. Curve numbers for irrigated croplands were assumed equal to those for managed grasslands under poor hydrologic conditions (which are expected to increase runoff and therefore emulate an irrigated field). For unconsolidated bare areas, CN values were matched with the fallow bare soil in NEH-630, as well for ESA bare areas LC type. For consolidated bare areas, CN values were averaged from impervious areas according to NEH-630. Zones that have dual hydrological soil groups were assumed to be undrained. The ESA CCI-LC dataset had a spatial resolution of 300 m, and it was resampled to 250 m using the nearest neighbor method to match the HYSOGs250m spatial resolution. We used two input datasets (HYSOGs250m and the resampled ESA CCI-LC) with three CN look-up tables (one for each ARC) to generate the curve number products at the 250 m resolution (GCN250).

### Curve number for various antecedent runoff conditions

**Fig. 2:**
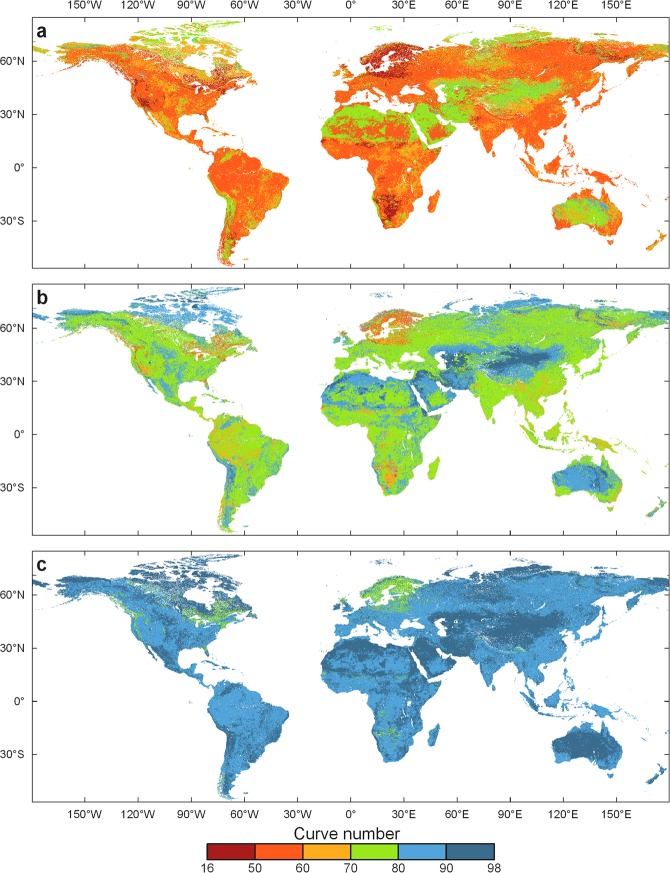
The three global curve number maps (GCN250). (**a**) Dry (ARCI), (**b**) average (ARCII), and (**c**) wet (ARCIII) antecedent runoff conditions.

The CN values vary depending on antecedent runoff conditions (ARC), which is affected by the rainfall intensity and duration, total rainfall, soil moisture conditions, cover density, stage of growth, and temperature^14^. For this reason, we generated three curve number maps for three ARCs: dry (Fig. 2a), average (Fig. 2b), and wet (Fig. 2c) ARC conditions. USDA^14^ provided guidelines to convert CN values from average ARC conditions into wet and dry ARC conditions.

## Data Records

**Fig. 3:**
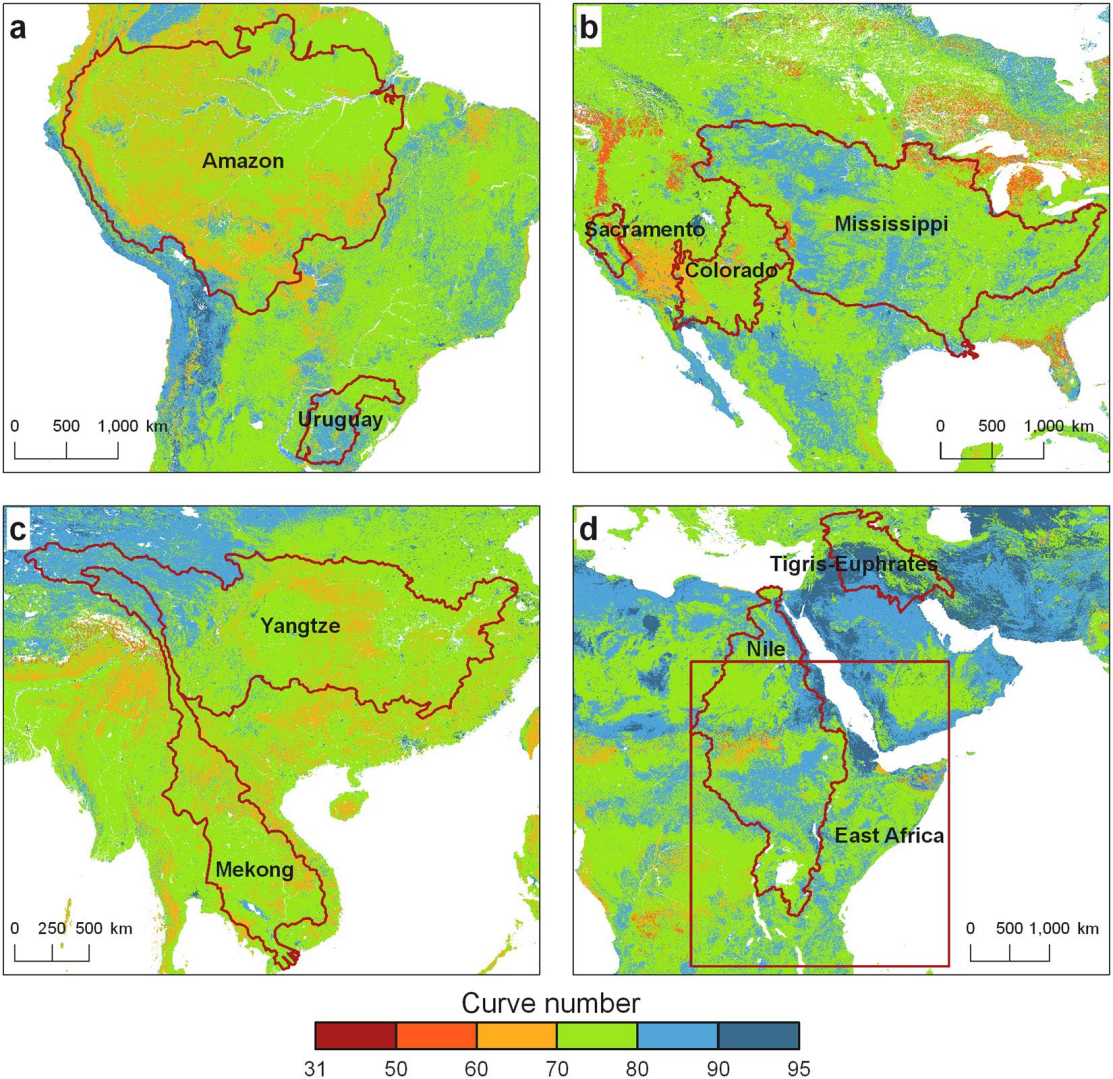
Curve number map (average antecedent runoff conditions) for selected river basins and regions around the world. (**a**) Amazon and Uruguay, (**b**) Colorado, Mississippi, and Sacramento (**c**) Mekong and Yangtze (**d**) Nile, Tigris-Euphrates, and East Africa.

There are three data records associated with this work: one raster dataset for each of the antecedent runoff conditions (ARCI = dry, ARCII = average, ARCIII = wet). The GCN250 product is publicly archived in Figshare^15^. The product is stored in GeoTiff format at 7.5 arc-second (~250 m spatial resolution) using the World Geodetic System 1984 (WGS84) datum geographic coordinate system. Table 2 presents a summary of the CN values mapping at the global and the continental scale. Figure 3 shows the distribution of the curve numbers for selected world river basin regions: (a) Amazon, (b) Mississippi, (c) Mekong, and (d) Nile and Tigris-Euphrates.

**Table 2:** Global and continental curve number (CN) values derived from GCN250.

Continent	CN (dry)				CN (average)				CN (wet)			
	min	max	µ	σ	min	max	µ	σ	min	max	µ	σ
Global	16	85	61.4	8.4	31	94	78.3	6.3	51	98	89.9	3.7
Africa	18	85	61.8	8.2	35	94	78.7	6.1	59	98	90.1	3.6
Asia	30	85	63.4	9.2	49	94	79.8	6.5	73	98	90.8	3.6
Australia	16	85	67.4	8.5	31	94	82.8	6.0	51	98	92.4	3.2
Europe	18	85	55.5	9.1	35	94	73.6	7.6	59	98	87.0	5.0
North America	18	85	60.7	9.4	35	94	77.8	7.2	59	98	89.6	4.4
South America	36	85	60.0	6.8	56	94	77.5	5.1	75	98	89.4	3.0
Oceania	36	85	60.8	7.3	56	94	78.0	5.6	75	98	89.8	3.3

Data is for three antecedent runoff conditions: average, dry, and wet. min is the minimum CN, max is the maximum CN, µ is the CN spatial mean, and σ is the CN spatial standard deviation.

## Technical Validation

The uncertainty of the GCN250 product is related to the uncertainty of the global ESA-CCI-LC dataset, the soils classification data, and the uncertainty in the CN look-up table. The compositing of CNs is affected by the accuracy of the Land cover classes, and the accuracy of hydrologic soil groups classifications in the HYSGOG250m database. Using gridded precipitation products from the Global Land Data Assimilation System (GLDAS)^16^, we compared the daily and the monthly runoff ratios resulting from the three GCN250 products with those of GLDAS runoff.

### Land cover

The ESA CCI-LC was evaluated by independent means where validation was carried out by external partiesfnline^9^. When validating the 2015 ESA CCI-LC product, the accuracy level was found to be between 71.1% and 75.4%. User accuracy was high (83–97%) for croplands (irrigated and rainfed), broadleaved evergreen forests, urban areas, bare areas, water bodies, and permanent snow and ice. Low user accuracies were observed for the classes of natural vegetation, lichens and mosses, sparse vegetation, flooded forest, and forests (mixed broadleaf and needle leaf). We expect curve numbers of these areas to have a higher uncertainty compared to other areas with better LC classification accuracy. Moreover, because the CN method was originally developed based on observation of rainfall-runoff relationships in small agricultural watersheds, we expect some uncertainty in curve numbers developed for forested watersheds in humid environments and deep soils. Hydrologists should proceed with caution when using these CNs for design and other hydrologic applications and should always compare generated runoff with observed values whenever possible.

### Hydrologic soil groups

Ross, *et al*.^8^ described the uncertainty assessment of the HYSOGs250m product that we used to generate the GCN250 dataset. The root means square error (RMSE) of the soil grids used as input to HYSOGs250m are between 9.5% and 13.1%. HYSOGs250 also included the groundwater table depth metric to capture broad-scale patterns of groundwater with a coefficient of variation of 9%. We expect these uncertainties to carry over into the GCN250 product.

### Comparison with other curve number datasets

Table 3 shows a comparison of CN values between our GCN250 and CN reported by Zeng, *et al*.^5^ for several large basins in the world. We believe that the (1–17%) higher values of composite GCN250 curve numbers compared to those reported in the Zeng, *et al*.^5^ under average ARC conditions for the studied basins to be mainly due to the difference in the soils input map. According to Zeng, *et al*.^5^, the global soil distribution is dominated by moderately low runoff potential (37% soil group B), whereas it is dominated by moderately high runoff (57% soil group C) according to Ross, *et al*.^8^, and therefore the resulting CNs are higher for our results due to the prevalence of higher runoff soils. The CNs are highly impacted by soil groups. For example, woods in poor conditions in a B hydrologic soil group would have a CN of 66, while the CN in a C hydrologic soil group would be 77, which doubles the runoff from say a 75 mm rainfall event. Furthermore, Ross, *et al*.^8^ factored the depth to impermeable layers (bedrock) and depth to the groundwater in the HYSOGs250m product, and both variables were absent from the Zeng, *et al*.^5^ study. Zeng, *et al*.^5^ used 17 classes from MODIS at 500 m resolution to generate the CN dataset, whereas we used the 36 classes of the 300-m ESA-CCI LC product to create the GCN250 product. We believe that the GCN250 dataset better captures land cover variations and soils hydrologic classifications than currently existing curve number datasets.

**Table 3:** Comparison of curve number (CN) values generated by GCN250 and CN reported in the literature for several basins.

Basin	Basin area (km^2^)	CN (Zeng, *et al*.^5^)	CN GCN250 (dry)	CN GCN250 (average)	CN GCN250 (wet)
Mississippi	5,649,686	72.0	59.3	77.0	89.3
Colorado	1,042,765	66.5	56.5	74.7	87.6
Sacramento	208,954	74.1	56.2	74.3	87.4
Yangtze	2,279,818	69.2	57.2	75.3	88.3
Mekong	905,344	69.2	57.7	75.8	88.6
Amazon	6,179,569	64.5	57.5	75.6	88.3
Uruguay	458,115	76.2	63.5	80.1	91.1

### Comparison of GCN250 runoff with GLDAS runoff

**Fig. 4:**
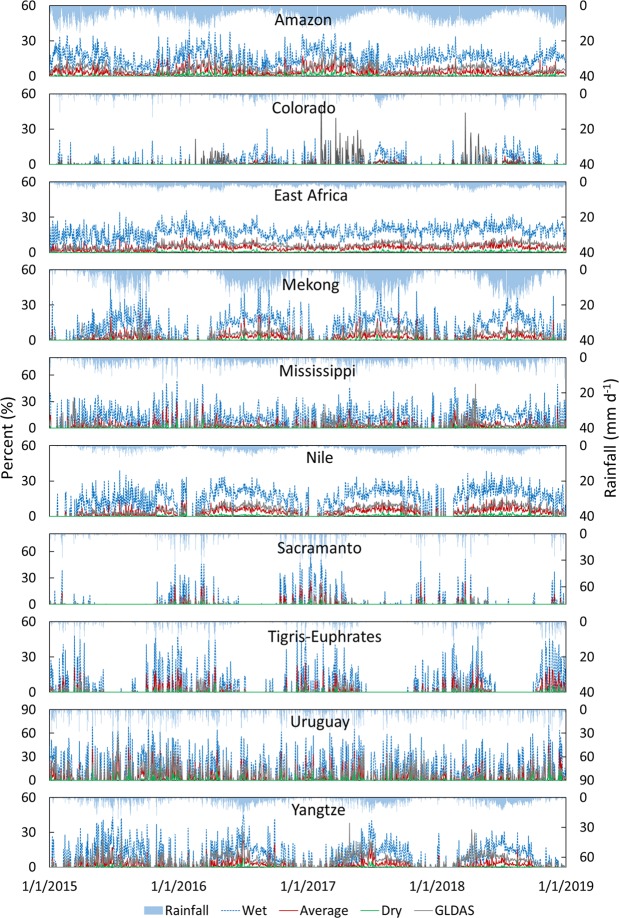
Comparison between GCN250 and GLDAS daily runoff ratios derived from GLDAS rainfall for major world basins and East Africa. Wet = GCN250 ARCIII runoff ratio, Average = GCN250 ARCII runoff ratio, Dry = GCN250 ARCI runoff ratio.

**Fig. 5:**
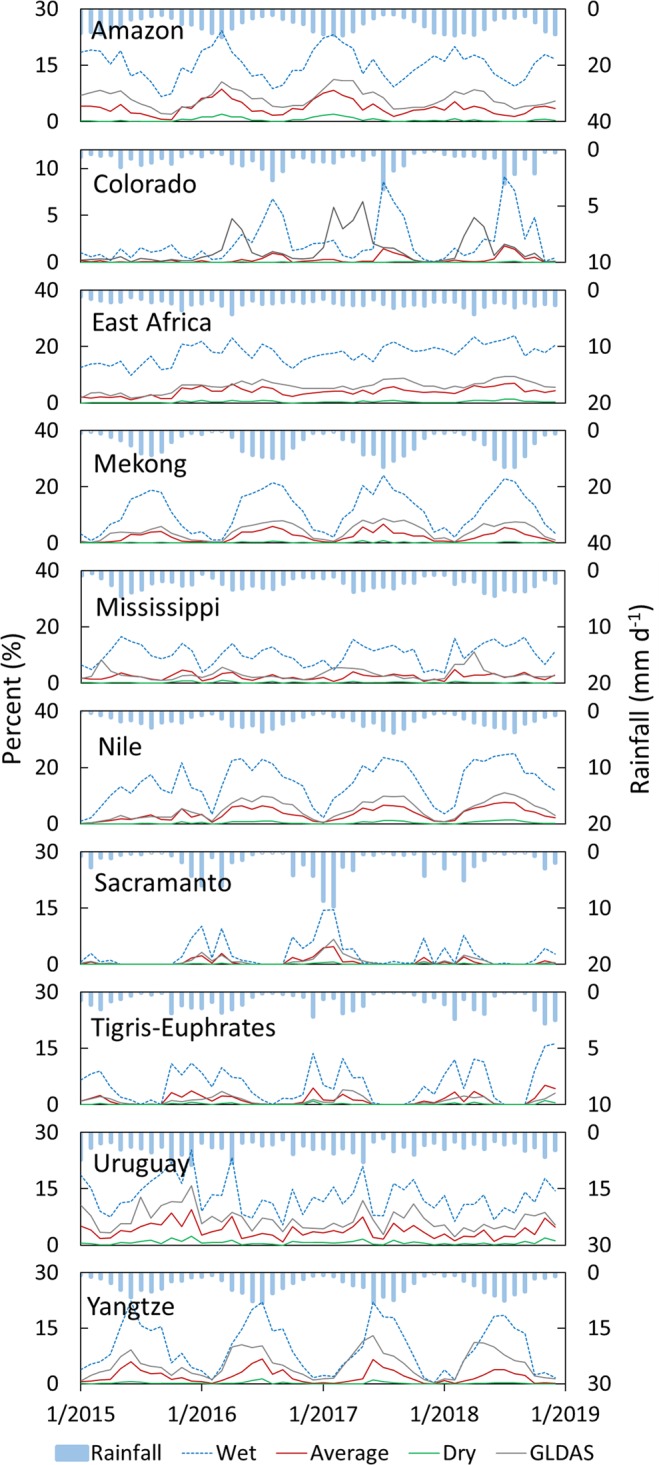
Comparison between GCN250 and GLDAS mean monthly runoff ratios derived from GLDAS rainfall for major world basins and East Africa. Wet = GCN250 ARCIII runoff ratio, Average = GCN250 ARCII runoff ratio, Dry = GCN250 ARCI runoff ratio.

We compared GCN250-generated runoff ratios to GLDAS runoff ratios (daily runoff/daily rainfall) for the following river basins: Amazon River Basin (South America), Colorado River basin (USA, Mexico), Mississippi (USA), Mekong River Basin (Cambodia, Vietnam, Thailand, Laos, Myanmar, China), Nile River Basin, Sacramento (USA), Tigris-Euphrates (Turkey, Iraq, Syria, Iran), Uruguay, Yangtze (China), also the area of East Africa (Fig. 3). Using daily gridded rainfall (aggregated from 3-hourly 0.25° × 0.25° rainfall) from the Global Land Data Assimilation System (GLDAS v2.1)^16^, we generated gridded daily runoff ratios from the GCN250 datasets (one for each of the three antecedent runoff conditions) and compared it to the aggregated daily runoff ratios (from the 3-hourly runoff) from GLDAS for 2015–2018. We applied the rainfall from GLDAS grid onto each CN pixel within that grid, generated the CN runoff, aggregated the runoff over the basin, and then calculated the mean runoff ratio by dividing the mean runoff by the mean rainfall over the basin for that day. We compared the time series of the daily mean runoff ratios from the two sets (GLDAS and GCN250) (populated over all the basins individually) (Fig. 4) and the mean monthly runoff ratios (Fig. 5). Results show that correlations between mean monthly GLDAS runoff ratios and GCN250 runoff ratios varied by basin and by rainfall. For example, good agreement between GLDAS runoff ratio and GCN250 average CN runoff ratio was noticed in the Nile and the Amazon basins and also (to a lower degree) the Tigris-Euphrates, while for Sacramento River basin, the GLDAS runoff ratio was in agreement with the GCN250 wet CN runoff ratio. For the Yangtze River basin, we noticed good agreement between GCN250 wet CN runoff ratios and GLDAS runoff ratios for December and April of every year. In summer, GLDAS runoff ratios ranged between the GCN250 average CN runoff ratio and the GCN250 wet CN runoff ratio. In the Mississippi basin, GCN250 average CN runoff ratio agreed with GLDAS runoff ratio during the May–December. For the winter months, the GLDAS ratios were closer to the GCN250 wet CN runoff ratio. Basins dominated by snowmelt (such as the Colorado River basin) showed temporal disagreement. We attribute this variability in the results mainly to the assumption of a constant average initial abstraction of 20% of the CN method and to the variability in the ARC conditions within a watershed in space and in time. We note that the objective of this exercise is not to validate the CN method against other direct runoff data, but rather to provide insights into how close the GCN250-based runoff is to GLDAS and possibly guidance on how the dataset can be used in different geographic and climatic conditions. Users are encouraged to check local conditions and runoff trends whenever available in their area of interest before deciding on which of the three GCN250 products (ARCI, ARCII, and ARCIII) to use. The best approach would be to utilize a combination of watershed land use knowledge, rainfall intensity, plant growth stage (for agricultural watersheds), antecedent moisture (from gridded soil moisture datasets), and precipitation products to determine the antecedent runoff conditions.

### ISA-Tab metadata file

Download metadata file

## Data Availability

The R script used for generating the GCN250 datasets is available for download^15^ with instructions for code reuse. The code can be used for generating CNs for future Land cover datasets.

## Online-only Table

**Online-only Table 1:** Curve numbers (CN) for hydrological soil groups (HSG) (A, B, C, and D) for ESA CCI land cover (LC) classes and their PFT decomposition percentages.

LC ID	LC Name	Tree				Shrub				Grass		Bare soil	Water	CN for HSG			
		Br. Ev.	Br. De.	Ne. Ev.	Ne. De.	Br. Ev.	Br. De.	Ne. Ev.	Ne. De.	Natural	Managed			A	B	C	D
0	No Data													NA	NA	NA	NA
10	Cropland, rainfed										100			57	68	75	78
11	Herbaceous cover										100			57	68	75	78
12	Tree or shrub cover						50				50			49	65	75	79
20	Cropland, irrigated or post‐flooding^a^										100			61	71	77	80
30	Mosaic cropland (>50%)/natural vegetation (tree, shrub, herbaceous cover) (<50%)	5	5			5	5	5		15	60			49	64	74	79
40	Mosaic natural vegetation (tree, shrub, herbaceous cover) (>50%)/cropland (<50%)	5	5			7.5	10	7.5		25	40			46	63	74	79
50	Tree cover, Br., Ev., closed to open (>15%)	90				5	5							31	56	70	77
60	Tree cover, Br., De., closed to open (>15%)		70				15			15				35	59	72	79
61	Tree cover, Br., De., closed (>40%)		70				15			15				35	59	72	79
62	Tree cover, Br., De., open (15‐40%)		30				25			35		10		42	63	75	81
70	Tree cover, Ne., Ev., closed to open (>15%)			70		5	5	5		15				35	59	72	79
71	Tree cover, Ne., Ev., closed (>40%)			70		5	5	5		15				35	59	72	79
72	Tree cover, Ne., Ev., open (15‐40%)			30			5	5		30		30		49	68	78	84
80	Tree cover, Ne., De., closed to open (>15%)				70	5	5	5		15				44	64	76	82
81	Tree cover, Ne., De., closed (>40%)				70	5	5	5		15				44	64	76	82
82	Tree cover, Ne., De., open (15‐40%)				30		5	5		30		30		53	70	80	85
90	Tree cover, mixed leaf type (Br. and Ne.)		30	20	10	5	5	5		15		10		41	62	75	81
100	Mosaic tree & shrub (>50%)/herbaceous cover (<50%)	10	20	5	5	5	10	5		40				37	60	73	79
110	Mosaic herbaceous cover (>50%)/tree & shrub (<50%)	5	10	5		5	10	5		60				38	60	73	80
120	Shrubland					20	20	20		20		20		48	66	77	83
121	Evergreen shrubland					30		30		20		20		48	65	77	83
122	Deciduous shrubland						60			20		20		48	67	78	83
130	Grassland									60		40		54	71	81	86
140	Lichens and mosses									60		40		54	71	81	86
150	Sparse vegetation (tree, shrub, herbaceous cover) (<15%)	1	3	1		1	3	1		5		85		71	82	88	92
152	Sparse shrub (<15%)					2	6	2		5		85		72	82	88	92
153	Sparse herbaceous cover (<15%)									15		85		71	82	88	92
160	Tree cover, flooded, fresh or brackish water	30	30							20			20	47	66	77	83
170	Tree cover, flooded, saline water	60				20							20	46	65	76	82
180	Shrub or herbaceous cover, flooded, fresh/saline/brackish water		5	10			10	5		40			30	57	72	82	86
190	Urban areas		2.5	2.5						15		75	5	70	82	88	91
200	Bare areas^b^											100		77	86	91	94
201	Consolidated bare areas^c^											100		85	90	93	94
202	Unconsolidated bare areas^b^											100		77	86	91	94
210	Water bodies												100	NA	NA	NA	NA
220	Permanent snow and ice													NA	NA	NA	NA
255	No Data													NA	NA	NA	NA

Br. = broadleaf; Ne. = needle leaf; Ev. = evergreen; De. = deciduous. A, B, C, and D are the hydrological soil groups; LC ID corresponds to Landcover ID in ESA-CCI LC dataset.

^a^CN values are the average of poor hydrologic conditions for all cropland types.

^b^CN values are from NEH-630 (fallow barren)’.

^c^CN values are the average of impervious areas from NEH-630.
